# Changes in the characteristics of Suicide Attempts during COVID-19 pandemic

**DOI:** 10.1192/j.eurpsy.2023.872

**Published:** 2023-07-19

**Authors:** J. Curto Ramos, N. Kishanchandani Chandiramani, M. Torrijos, J. Andreo-Jover, B. Orgaz-Alvarez, M. Velasco, D. García Martínez, G. Juárez, S. Cebolla, P. Aguirre, B. Rodríguez Vega

**Affiliations:** 1 Department of Psychiatry, Clinical Psychology and Mental Health, La Paz University Hospital, Madrid, Spain, Hospital la Paz; 2 Faculty of Medicine, Universidad Autónoma de Madrid (UAM), Madrid, Spain, Madrid, Spain

## Abstract

**Introduction:**

Different studies indicate high prevalence’s of suicidal behaviour, anxiety, depression, insomnia, and PTSD associated with the COVID-19 pandemic. There is currently not enough scientific evidence available to analyze the impact that the COVID-19 pandemic has had on the rate of suicide attempts and their characteristics.

**Objectives:**

To analyze and compare the characteristics of suicidal behavior (in terms of method, severity, medical damage produced and need for hospitalization) of patients attended during the COVID-19 pandemic compared to previous years.

**Methods:**

A retrospective study was performed based on a standardized data collection of patients attending the University Hospital La Paz between April 2018 and November 2021. 581 patients who attempted suicide at least once were included in this study. We compared the severity using the Beck Suicide Intent Scale. Chi-square ant Student’s t were used to compare clinical characteristics such as medical damage, method of suicide attempt and indication for admission after the attempt, between suicide attempts during the COVID-19 pandemic and previous years.

**Results:**

Our results suggest that during the COVID-19 pandemic suicide attempts caused more medical damage (p<0.001), had higher severity (p<0.000), and required more admission in Intensive Care Units, General Internal Medicine and Psychiatry compared with pre-Covid years (p<0.000).

**Conclusions:**

This is the first study in Spain analysing the changes in characteristics of suicide attempts during the COVID-19 pandemic. This has important implications for reducing suicide rates, preventing future attempts, and enabling us to design specific treatments of Suicidal Behaviour.

**Disclosure of Interest:**

None Declared

